# Medical and Physical Intelligence

**Published:** 1803-02-01

**Authors:** 


### MEDICAL AND PHYSICAL INTELLIGENCE.

### JENNEREAN INSTITUTION.

A Society has just been formed in London for the universal extension of the Vacciolous Inoculation, of the progress of which we propose to present our Readers with the earliest information, as it promises to become an object even of national pride as well as of incalculable utility, from the spirit with which it has been instituted, and the interest which some of the most eminent or worthy characters offer to take in its Establishment.

At a very numerous and highly respectable meeting, holden at the London Tavern, on Wednesday, Jan. 19, 1803, to consider of the best means to be adopted for the extermination of the Small-Pox, the Right Hon. the Lord Mayor in the Chair; the following Address was read, and presented to the Chair by Benjamin Travers, Esq.

"Address to the Public.

"The dreadful havock occasioned by that horrid pestilence, the Small-pox, which, in the united kingdom alone, annually sweeps away more than 40,000 persons, has long been a subject of deep regret to every humane and reflecting mind.

"The inoculation of this disease has opposed an ineffectual resistance to its destructive career. Although, confessedly, a valuable improvement in rendering the disease more mild, yet such has been the consequence of the partial adoption of the practice, that it appears, on a careful review of the history of the Small-pox, that inoculation, by spreading the contagion, has considerably increased its mortality.

"A new species of inoculation has at length been providentially introduced by our countryman, Dr. Jenner, which, without being contagious, without occasioning any material indisposition, or leaving any blemish, proves an effectual preservative against the Small Pox.

"The House of Commons having investigated this subject with the most scrupulous attention, and being perfectly convinced of the superior advantages resulting from this discovery, have given their sanction to the practice; the safety, mildness, and efficacy of which more than half a million of instances have fully confirmed.

"The unspeakable benefits which may be expected to arise from an extensive diffusion of this salutary practice, will be much accelerated by the establishment of an Institution in the central part of the Metropolis, on a broad basis, supported with a spirit equal to the design, and worthy of the character of the British nation.

And

And when the magnitude of the object is considered, which is no less than to eradicate a disease, acknowledged to be the greatest scourge that ever afflicted mankind, there can be but one sentiment on the subject.

"The enlightened, the benevolent, the opulent, will doubtless vie with each other in the zealous support of an undertaking which will reflect the highest honour upon their country; and by saving millions of victims from an untimely grave, prove an inestimable blessing to the whole human race."

The Right Hon. the Lord Mayor having read the Address from the chair, it was moved by Dr. Letsom, and seconded by Dr. Bradley, 'That the Address be received and adopted." Which was carried unanimously.

A Motion having been made by Mr. Gurney, and seconded by Joseph Leaper, 'That this meeting do form itself into a Society for the extermination of the Small-pox'—

The Hon. Admiral Berkley arose, and said, 'that he had it in command from his Royal Highness the Duke of Clarence to apologize to the meeting for his non-attendance, he having been unavoidably prevented from doing himself the pleasure and the honour of attending on the present interesting occasion; but that his Grace the Duke of Bedford held a motion in his hand, which, had his Royal Highness been present, he himself would have made.'

On this the Right Honourable Chairman observed, that a motion had already been made and seconded; and that, consequently, it must first be disposed of.

The Hon. Admiral acknowledged the propriety of the observation, but said that the motion was proposed as a tribute of esteem to a Benefactor of the World, and that if the previous motion could, for the moment, be waved, and the wish of his Royal Highness be acceded to, the honour intended, if voted, would be greater coming from a popular assembly, than if it were conferred by any organised society.

Mr. Gurney, on this, immediately offered priority to the wish of his Royal Highness; when,

On the motion of his Grace the Duke of Bedford, at the special request of his Royal Highness the Duke of Clarence, seconded by the Hon. Admiral Berkeley—It was

Resolved unanimously, That the thanks of this meeting be transmitted to Dr. Jenner, expressive of the high sense which it entertains of his merit, and the great importance of his discovery; and, particularly, for the liberal offer of his assistance to accomplish the great object it has in view.

Mr. Gurney's motion, That this meeting do form itself into a Society for the Extermination of the Small-pox was then carried unanimously.

On the motion of J. J. Angerstein, Esq.—It was

Resolved unanimously, That a subscription be now opened, to prosecute the laudable intentions of this Society.

Resolved unanimously, That the following three gentlemen be appointed Trustees, viz. the Right Hon. the Lord Mayor, John Julius Angerstein, Esq. and Benjamin Travers, Esq.

Resolved unanimously, That a Committee be now appointed for considering of and forming a Plan for the purpose of carrying into effect the important object of this Society, with liberty to add such gentlemen as they may think proper; that five be a quorum, and that they report the same to another General Meeting to be called by them.

The Committee having been nominated, it was

Resolved unanimously, That the thanks of this Society be given to the Governors and Officers of the Small-pox Hospital, for their very liberal offer to co-operate in the purposes of this Society.

Resolved unanimously, That the thanks of this Society be given to the Right Hon. the Lord Mayor, for his respectful attention, cordial support, and able conduct in the Chair.

Resolved unanimously, That all the Bankers of London, Westminster, and Southwark, and the Members of the Committee, be requested to receive subscriptions.

*** About 1000*l.* has been already subscribed to the Institution, and the Committee are zealously employed in giving effect and activity to the Resolutions.

---

### SMALL-POX AND INOCULATION HOSPITALS, PANCRAS.

Extract from the Committee's Report presented to a General Court, at Batson's Coffee-House, London, on the 16th December, 1802.

"Your committee, in addition to what they stated in their last report, desire to recal to your attention the increased public benefit of this Institution, since the introduction of Vaccine Inoculation has been added to the former branches of its practice. It began in this hospital, under the direction of Dr. Woodville, in Jan' 1799; and from that period to the 1st of December instant, 11,800 patients and upwards have been vaccinated, of which number about 2,500 were afterwards proved to be secure from the *Natural Small-Pox*, by receiving a further inoculation according to the former practice, which took no effect; a number amply sufficient to satisfy the public mind of the security and success of the new practice of Vaccination. And your committee add, that they have not heard of any complaint from any one of those who were not inoculated a second time, of their having since taken the natural Small-pox, although they were chiefly indigent persons, and the far greater number of them living in places where the air is very confined, and particularly where it has been since ascertained that the natural Small-Pox was prevalent amongst those with whom many of them necessarily had continual intercourse.

"The success of Vaccination has very rapidly increased during the current year. From the 1st of January last to the 1st of

December

December instant, of 373 patients admitted into the Inoculation Hospital, only 49 were inoculated according to the former practice; and of 4,005 out-patients, only 39 were inoculated according to the former practice; whereby it appears that the relief of the Institution has been afforded to 4,378 patients by Inoculation, of whom 88 having been inoculated according to the former practice, the blessing of Vaccination has been extended to 4,290 persons in eleven months by this Hospital. One hundred and fifty-nine patients in the natural Small-pox have been received into that department of this Institution; and medicines and advice have been granted to 55 children also in that disease, who were brought to the hospital as out-patients.

"This very extensive practice, under the skilful management of Dr. Woodville, the physician, and of Mr. Wachsell, the resident surgeon, have enlarged the sphere of this Institution, rendered it more beneficial to the poor, and increased its claim upon the public liberality.

"That liberality which has ever fostered its beneficent designs, has enabled your committeee to enjoy the lively satisfaction of co-operating in its promotion, and of witnessing the numerous instances of its general good.

"A. HIGHMORE, Sec."

31l. 10s. constitute a Governor for life, and 5l. 5s. an annual Governor; and other donations are gratefully received."

---

Professor ALDINI, of the University of Bologna, availed himself of the opportunity afforded by the execution of Forster, on the 27th inſt. for the murder of his wife and child, to repeat his experiments on the theory of his uncle Galvani. A liberal offer had been made him of the use of that subject by Mr. Keate, surgeon to the king, who was himself present on this occasion. The result of this experiment promises the greatest advantages to the intereſts of humanity, especially in cases of apparent death by drowning, and other cases of asphyxia.

We hope that Mr. Keate and the Professor will shortly gratify the public with a particular account of an experiment, the first of its kind in Great Britain. The corpse was made to exhibit very powerful muscular contractions before dissection, and that afterwards these contractions continued for seven hours and a half. On the first application of the process to the face, the jaw of the deceased criminal began to quiver, and the adjoining muscles were horribly contorted, and one eye was actually opened. In the subsequent part of the process, the right hand was raised and clenched, and the legs and thighs were set in motion. It appeared to the uninformed part of the bye-ſtanders, as if the wretched man was on the eve of being restored to life.

During his residence in England, Professor Aldini exhibited his experiments at Oxford, at Mr. Wilson's Anatomical Theatre in London, and at St. Thomas's and Guy's Hospitals. And we learn with with pleasure, that the lecturers and pupils of these two Hospitals have presented him with a gold medal, in honourable testimony of their approbation.

---

A Translation is in forwardness, and will speedily be published, of SUE's History of Galvanism, to which will be annexed a variety of copper-plates, and a familiar Introduction to the practice of that curious and interesting Science.

---

*Humours of the Eye.*—Mr. CHENEVIX has analyfed the humours of the eye, making his experiments upon the eyes of sheep, and he considers the aqueous and vitreous humours as composed of water, albumen, gelatine, and muriate of soda: the chrystalline contains no muriate of soda.

---

A Gentleman at Baltimore, in America, in a letter to Dr. PATTERSON, of Londonderry, states, "that the Vaccine Inoculation goes on with success all over that Continent. To the *Southern* States, it is a blessing beyond all price, as even the inoculation for the Small-pox was a business of difficulty and danger; yet their physicians were extremely slow in adopting the discovery. This year, however, they have begun with great spirit, and the practice is become universal. That most amiable man, the prefent President of the United States, with his usual philanthropy, early used all his endeavours to introduce into Virginia, "*this first best Gift of Heaven.*"

---

Mr. HUGUENIN, No. 33, Haymarket, has opened a warehouse for the genuine Lichen Islandicus and the preparations of it, recommended by Dr. Regnault, in the united quality of nourishment and medicine, conducted according to his prescriptions, and under his direction.

---

Mr. POWER's new Work, entitled Observations on the Ophthalmia of Egypt, will be published in the course of a few days.

---

Mr. THOMAS will commence his Spring Courfe of Lectures on the Practice and Operations of Surgery, on Wednesday, February 2, at seven o'clock in the evening, at his House in Leicester Square.

